# Evaluation of the Hitachi 717 analyser

**DOI:** 10.1155/S1463924689000349

**Published:** 1989

**Authors:** Carmen Biosca, Felipe Antoja, Cristina Sierra, Helene Douezi, Magda Macià, María-Jesús Alsina, Román Galimany

**Affiliations:** 1 Servei d'Anàlisis Clíniques Hospital Germans Trías i Pujol Carretera de Canyet s/n Badalona Barcelona 08916 Spain; 2 Servei d'Anàlisis Clíniques Centre d'Assistència Primària Dr. Robert Plaça de la Medicina s/n Badalona Barcelona 08915 Spain

## Abstract

The selective multitest Boehringer Mannheim Hitachi 717 analyser was evaluated according to the guidelines of the Comisión de Instrumentación de la Sociedad Española de Química Clinica and the European Committee for Clinical Laboratory Standards. The evaluation was performed in two steps: examination of the analytical units and evaluation in routine operation.

The evaluation of the analytical units included a photometric study: the inaccuracy is acceptable for 340 and 405 nm; the imprecision ranges from 0.12 to 0.95% at 340 nm and from 0.30 to 0.73 at 405 nm, the linearity shows some dispersion at low absorbance for NADH at 340 nm, the drift is negligible, the imprecision of the pipette delivery system increases when the sample pipette operates with 3 μl, the reagent pipette imprecision is acceptable and the temperature control system is good.

Under routine working conditions, seven determinations were studied: glucose, creatinine, iron, total protein, AST, ALP and calcium. The within-run imprecision (CV) ranged from 0.6% for total protein and AST to 6.9% for iron. The between run imprecision ranged from 2.4% for glucose to 9.7% for iron. Some contamination was found in the carry-over study. The relative inaccuracy is good for all the constituents assayed.


Journal of Automatic Chemistry Vol. 11, No. 4 (July–August 1989), pp. 159–163

### Evaluation of the Hitachi 717 analyser

**Carmen Biosca**

*Servei d'Anàlisis Clíniques, Hospital Germans Trías i Pujol, Carretera de Canyet s/n, 08916 Badalona, Barcelona, Spain*

**Felipe Antoja**

*Servei d'Anàlisis Clíniques, Centre d'Assistència Primària Dr. Robert, Plaça de la Medicina s/n, 08915 Badalona, Barcelona, Spain*

**Cristina Sierra and Helene Douezi**

*Servei d'Anàlisis Clíniques, Hospital Germans Trías i Pujol, Carretera de Canyet s/n, 08916 Badalona, Barcelona, Spain*

**Magda Macià and María-Jesús Alsina**

*Servei d'Anàlisis Clíniques, Centre d'Assistència Primària Dr. Robert, Plaça de la Medicina s/n, 08915 Badalona, Barcelona, Spain*

**Román Galimany**

*Servei d'Anàlisis Clíniques, Hospital Germans Trías i Pujol, Carretera de Canyet s/n, 08916 Badalona, Barcelona, Spain*

*The selective multitest Boehringer Mannheim Hitachi 717 analyser was evaluated according to the guidelines of the Comisión de Instrumentación de la Sociedad Española de Química Clínica and the European Committee for Clinical Laboratory Standards. The evaluation was performed in two steps: examination of the analytical units and evaluation in routine operation.*

*The evaluation of the analytical units included a photometric study: the inaccuracy is acceptable for 340 and 405 nm; the imprecision ranges from 0·12 to 0·95% at 340 nm and from 0·30 to 0·73 at 405 nm, the linearity shows some dispersion at low absorbance for NADH at 340 nm, the drift is negligible, the imprecision of the pipette delivery system increases when the sample pipette operates with 3 µl, the reagent pipette imprecision is acceptable and the temperature control system is good.*

*Under routine working conditions, seven determinations were studied: glucose, creatinine, iron, total protein, AST, ALP and calcium. The within-run imprecision (CV) ranged from 0·6% for total protein and AST to 6·9% for iron. The between run imprecision ranged from 2·4% for glucose to 9·7% for iron. Some contamination was found in the carry-over study. The relative inaccuracy is good for all the constituents assayed.*

### Introduction

The Boehringer Mannheim Hitachi 717 is a selective multichanel analyser with a theoretical throughput of 750 tests per hour (with electrolytes, during routine operation). The evaluation reported here was carried out according to the guidelines of the European Committee for Clinical Laboratory Standards (ECCLS) [1] and the protocol of the Comisión de Instrumentación de la Sociedad Española de Química Clínica (SEQC) [2].

The evaluation of the analytical units included a photometric study: inaccuracy, imprecision, drift and linearity, together with the imprecision of the pipette delivery system and the temperature control system. Imprecision (within- and between-run), carry-over and relative inaccuracy were studied under routine working conditions.

Seven determinations, creatinine, total protein, alanine aminotransferase, glucose, alkaline phosphatase, iron and calcium, were chosen in order to cover nearly all performance criteria of the instrument. The relative inaccuracy was studied in comparison with the results obtained with Ultrolab-Aurora and Technicon/RA-1000 analysers.

### Materials and methods

*Instrument*

The Hitachi 717 (manufactured by Boehringer Mannheim, Mannheim, FRG) is a discrete, selectively operated multitest analyser, which can perform up to 35 different tests on one sample. Three tests, sodium, potasium and chloride, are performed by ion-selective electrodes (optional unit).

The routine procedure can be interrupted by stat samples at any time, returning afterwards to the original sequence. Enzyme and substrate determinations can be measured by the instrument and also by non-linear chemistries.

The individual reagents are pipetted directly into the plastic reaction cuvette, incubated and read mono- or bichromatically by a diffraction-grating photometer with 12 fixed wavelengths. The sample pipetter allows a smooth adjustment to any volume between 3 and 20 µl, adjustable in steps of 1 µl. Subsequently pipetting of two different reagents can be performed. The range of the reagent dispensing volume is 250–350 µl.

The Hitachi 717 is microprocessor-controlled. It enables the operator to have a clear overview of the mechanics, electronics and chemistries at any time.

*Reagents*

Non-standard abbreviations: BM, Boehringer Mannheim; TCN, Technicon; K, Knickerbocker; B, Behring; AST, aspartate aminotransferase (E.C. 2.5.1.2); ALP, alkaline phosphatase (E.C. 3.1.3.1); DEA, diethanolamine buffer; PNP, *p*-nitrophenol; NADH, β-nicotinamide adenine dinucleotide, reduced form; NaOH, sodium hydroxide; TPTZ, tripyridyltriazine; C. L., confidence limits.

For the evaluation of the analytical units: PNP 709·5 µmol $l^{-1}$ (Knickerbocker E 502); NaOH Merck 6498; from a solution of 0·36 mmol $l^{-1}$ of PNP in NaOH (20 mmol $l^{-1}$) different concentrations were obtained by dilution; NADH disodium salt (Sigma N8129); tris(hydroxymethyl)methylamine (Merck 8382); all the other solutions were prepared from a 1 mmol $l^{-1}$ solution of NADH in 80 mmol $l^{-1}$ Tris.

0142–0453/89 $3.00 © 1989 Taylor & Francis Ltd.

For test on samples of sera: glucose (BM 1040863) (hexokinase); creatinine (BM 1040847) (Jaffé, without deproteinization); iron (BM 1040880) (ferrozine without deproteinization); total protein (BM 1040901) (biuret); AST (BM 1040740) (IFCC); ALP (BM 1040669) (DEA); calcium (BM 1040812) (cresolphthalein).

For comparison studies we used the following instruments (in routine use) and reagents. For correlation with the Technicon RA-1000 (Technicon Instruments, Tarrytown, NY, USA): glucose (TCN-T01-1833) (hexokinase); creatinine (TCN-T01-1927) (Jaffé without deproteinization); AST (K cod. E 532) (Tris, IFCC); total protein (K cod. B 259) (biuret); calcium (K cod. B 270) (methylthymol blue). For correlation with the Ultrolab-Aurora (Ultrolab, Stockholm, Sweden): iron (B, AU 407) (TPTZ, with serum blank and without deproteinization); ALP (B, OURN 30) (DEA). For the calibration: BM 759350.

*Table 1. Photometric inaccuracy.*

| | Theoretical absorbance | Mean observed absorbance | Inaccuracy (%) |
|---|---|---|---|
| NADH (340 nm) | 0·400 | 0·381 | −4·8 |
| | 0·800 | 0·751 | −6·1 |
| | 1·200 | 1·109 | −7·6 |
| | 1·600 | 1·495 | −6·5 |
| | 1·997 | 1·872 | −6·2 |
| | 2·197 | 2·035 | −7·4 |
| PNP (405 nm) | 0·500 | 0·502 | +0·4 |
| | 1·000 | 0·997 | −0·3 |
| | 1·500 | 1·484 | −1·0 |
| | 2·000 | 1·921 | −3·9 |

*Table 2. Photometric imprecision.*

| | Mean absorbance | CV (%) |
|---|---|---|
| NADH (340 nm) | 0·052 | 0·95 |
| | 0·186 | 0·22 |
| | 0·448 | 0·19 |
| | 0·907 | 0·12 |
| | 1·795 | 0·18 |
| PNP (405 nm) | 0·066 | 0·73 |
| | 0·250 | 0·44 |
| | 0·639 | 0·47 |
| | 1·270 | 0·41 |
| | 2·447 | 0·30 |

### *Parameters evaluated*

*Photometric inaccuracy.* Photometric inaccuracy was studied at 340 nm with a solution of disodium NADH (333 μmol $l^{-1}$) in Tris buffer (80 mmol $l^{-1}$), and at 405 nm with PNP solution (143·8 μmol $l^{-1}$) in NaOH (20 mmol $l^{-1}$). Dilutions were prepared manually. Up to three consecutive measurements were made for each absorbance, using the same cuvette.

Inaccuracy was calculated from the experimental values and the theoretical values obtained from the molar absorptivities of NADH and PNP.

*Photometric imprecision.* From solutions prepared as previously, 30 successive measurements were obtained in the same cuvette, and from these were calculated the mean, standard deviation and coefficient of variation at both 340 and 405 nm.

*Photometric linearity.* Using serial dilutions prepared as before, three successive determinations were made for each absorbance, always in the same cuvette. Theoretical absorbances were calculated from the molar absorptivities of NADH and PNP.

*Photometric drift.* Photometric stability was studied over the first 30 min and at 12 h at 405 nm with PNP in NaOH solution of theoretical absorbance 1·000. Three successive determinations were made in the same cuvette.

*Sample pipette delivery system imprecision.* The reagent 1 delivery system was set to dispense a constant amount (250 μl) of NaOH (20 mmol $l^{-1}$), and the sampler (sample pipette) to dispense volumes ranging from 3 to 20 μl of PNP solution. In each case, the final absorbance was

*Table 3. Photometric linearity.*

| | NADH (340 nm) | | PNP (405 nm) | |
|---|---|---|---|---|
| Theoretical absorbance | Mean observed absorbance | Dispersion (%) | Mean observed absorbance | Dispersion (%) |
| 0·050 | 0·065 | +30·0 | 0·052 | +4·0 |
| 0·100 | 0·084 | −16·0 | 0·097 | −3·0 |
| 0·200 | 0·186 | −0·7 | 0·196 | −2·0 |
| 0·400 | 0·328 | −4·6 | 0·405 | +1·2 |
| 0·600 | 0·602 | +0·3 | 0·614 | +2·3 |
| 0·800 | 0·750 | −6·2 | 0·824 | +3·0 |
| 1·000 | 0·935 | −6·5 | 1·050 | +0·1 |
| 1·200 | 1·107 | −7·7 | 1·162 | −3·1 |
| 1·400 | 1·310 | −6·4 | 1·371 | −2·0 |
| 1·600 | 1·491 | −6·8 | 1·595 | −0·3 |
| 1·800 | 1·679 | −6·8 | 1·748 | −2·8 |
| 2·000 | 1·873 | −6·4 | 1·928 | −3·6 |

arranged to be around 0·500. The standard deviation and coefficient of variation were calculated from 30 determinations.

*Reagent pipettes delivery system imprecision.* The sampler pipette was blocked and, in a first experiment, the reagent 1 pipette dispensed volumes ranging from 65 to 350 μl of PNP solution whereas the reagent 2 pipette was set to dispense a constant amount (200 μl) of NaOH (20 mmol $l^{-1}$). A second experiment was carried out with volumes between 50 and 125 μl of PNP solution for the reagent 2 pipette and a constant amount (200 μl) of NaOH (20 mmol $l^{-1}$) for the reagent 1 pipette.

*Table 4. Imprecision of the sample pipette delivery system.*

| Sample pipette (μl PNP) | Reagent pipette (μl NaOH) | CV (%) |
|---|---|---|
| 3 | 250 | 2·10 |
| 9 | 250 | 0·71 |
| 11 | 250 | 0·78 |
| 14 | 250 | 0·83 |
| 20 | 250 | 0·74 |

*Table 5. Imprecision of the reagent pipettes delivery system.*

| Reagent pipette 1: PNP (μl) | Reagent pipette 2: NaOH (μl) | CV (%) |
|---|---|---|
| 65 | 200 | 0·42 |
| 160 | 200 | 0·95 |
| 208 | 200 | 0·53 |
| 255 | 200 | 0·44 |
| 350 | 200 | 0·91 |
| 200 | 50 | 1·09 |
| 200 | 75 | 0·84 |
| 200 | 88 | 0·90 |
| 200 | 100 | 1·22 |
| 200 | 125 | 1·08 |

In both experiments, the standard deviation and coefficient of variation were calculated from 30 successive determinations.

*Temperature control*

The warm-up time was studied making readings every 10 s, until three consecutive readings with a deviation of ±0·1 °C were obtained. Thirty readings were then made at 20-s intervals for 10 min. The mean, variance and coefficient of variation were calculated.

For evaluation of the system under working conditions, the parameters studied were as follows.

*Imprecision*

Within the same run, thirty samples of control sera were tested at three levels, in order to study the within-run imprecision. To evaluate the between-run imprecision a further thirty samples were distributed in different runs.

*Specimen-independent carry-over*

All combinations of method sequences were checked in order to study the reagent probe carry-over, using a pool of specimens in a pre-determined sequence run on three different days. The carry-over effect measured was compared with twice the within-run imprecision of the

*Table 6. Within- and between-run imprecision for concentrations and enzyme activities (mean ± SD) of some analytes measured with the Hitachi 717.*

| | | Within-run ($n$ = 30) | | Between-run ($n$ = 30) | |
|---|---|---|---|---|---|
| | | $\bar{x}$ ± SD | CV (%) | $\bar{x}$ ± SD | CV (%) |
| Glucose (mmol/$l^{-1}$) | H | 14·5 ± 0·10 | 0·7 | 14·4 ± 0·34 | 2·4 |
| | M | 4·7 ± 0·05 | 1·1 | 4·9 ± 0·15 | 3·1 |
| | L | 3·2 ± 0·04 | 1·2 | 3·3 ± 0·09 | 2·7 |
| Creatinine (mmol $l^{-1}$) | H | 689 ± 7·7 | 1·1 | 693 ± 31·7 | 4·6 |
| | M | 152 ± 2·1 | 1·4 | 153 ± 7·2 | 4·7 |
| | L | 97 ± 1·3 | 1·3 | 105 ± 4·6 | 4·3 |
| Iron (μmol $l^{-1}$) | H | 31·0 ± 0·6 | 1·9 | 32·0 ± 2·0 | 6·4 |
| | M | 20·4 ± 0·5 | 2·4 | 21·8 ± 2·0 | 9·0 |
| | L | 14·6 ± 1·0 | 6·9 | 15·2 ± 1·5 | 9·7 |
| AST (U $l^{-1}$) | H | 163·0 ± 1·1 | 0·7 | 168·0 ± 4·7 | 2·8 |
| | M | 36·0 ± 0·2 | 0·6 | 36·7 ± 1·4 | 3·9 |
| | L | 24·7 ± 0·4 | 1·6 | 25·1 ± 1·0 | 4·1 |
| ALP (U $l^{-1}$) | H | 188·0 ± 2·5 | 1·3 | 197·0 ± 6·3 | 3·2 |
| | M | 96·0 ± 1·2 | 1·3 | 101·0 ± 5·3 | 5·3 |
| | L | 68·0 ± 0·9 | 1·3 | 68·0 ± 3·7 | 5·4 |
| Total protein (g $l^{-1}$) | H | 71·5 ± 0·6 | 0·8 | 73·5 ± 2·5 | 3·4 |
| | M | 48·0 ± 0·3 | 0·6 | 50·3 ± 2·2 | 4·4 |
| | L | 34·0 ± 0·4 | 1·2 | 34·7 ± 2·1 | 3·4 |
| Calcium (mmol $l^{-1}$) | H | 2·78 ± 0·03 | 1·2 | 2·90 ± 0·11 | 3·8 |
| | M | 2·14 ± 0·05 | 2·3 | 2·22 ± 0·11 | 4·9 |
| | L | 1·42 ± 0·04 | 2·8 | 1·48 ± 0·07 | 4·7 |

method in question [1]. Washing unit-related carry-over was also studied.

*Sample-related carry-over*

Following a permutation order, two control samples with different concentrations were distributed along the sample disk. Three high specimens followed by three low specimens were processed and the carry-over ratio ($k$) was calculated. A mean value for ten determinations of $k$ was obtained [3].

*Method comparison with patients' specimens*

We analysed 100 fresh human sera (in different analytical series) covering the entire analytical range for each of the seven analytes, with the Hitachi 717 and the RA-1000 and Ultrolab-Aurora comparison instruments. The statistical evaluation was done by a non-parametric method of Passing and Bablok [4, 5].

### Results and discussion

*Photometric inaccuracy*

The photometric inaccuracy for NADH solution at 340 nm, expressed as percentage accuracy, was −7·4% for 2·035 absorbance and −4·8% for 0·381 absorbance. For PNP solution at 405 nm it was −3·9% for 1·921 absorbance and +0·4% for 0·502 absorbance. The photometric inaccuracy is acceptable for both 340 and 405 nm (see table 1).

*Photometric imprecision*

The photometric imprecision increases with decreasing absorbance, but the coefficients of variation are never as high as 1·0%; they ranged from 0·12 to 0·95% at 340 nm and from 0·30 to 0·73% at 405 nm (see table 2).

*Table 7. Sample-related carry-over.*

| | Concentration | | |
|---|---|---|---|
| | High | Low | $\bar{k}$ ($n = 10$) |
| Glucose (mmol $l^{-1}$) | 29·8 | 6·0 | +0·400 |
| Creatinine (μmol $l^{-1}$) | 1257 | 78 | +0·008 |
| Iron (μmol $l^{-1}$) | 63 | 15 | −3·044 |
| AST (U $l^{-1}$) | 851 | 22 | −0·048 |
| ALP (U $l^{-1}$) | 2164 | 181 | −0·014 |
| Total protein (g $l^{-1}$) | 131 | 70 | −0·248 |
| Calcium (mmol $l^{-1}$) | 5·7 | 2·3 | −0·290 |

*Photometric linearity*

The linearity obtained is correct for NADH at 340 nm and for PNP at 405 nm. The results are shown in table 3. There is some dispersion at low absorbances for NADH at 340 nm.

*Photometric drift*

For the photometric stability test, PNP was read at 405 nm for 30 min, using a solution with a mean absorbance of 1·170. The coefficient of variation obtained was 0·38%. With the same solution read at 12 h, the mean absorbance was 1·182 and the coefficient of variation was 0·66%. The drift is negligible.

*Sample pipette delivery system imprecision*

The sample pipette imprecision is acceptable, although it is directly related to the volume dispensed; it increases when the sample pipette is operated with 3 μl (see table 4).

*Reagent pipettes delivery system imprecision*

The reagent pipettes imprecision is acceptable. It ranged from 0·42 to 0·95% for reagent 1 pipette and from 0·84 to 1·22% for reagent 2 pipette (see table 5).

*Temperature control*

Testing each 10 s, the warm-up time to reach 37 °C was 12 min. An additional time of 3 min 40 s must be considered for initialization of the device.

The main temperature attained was 36·8 °C and the coefficient of variation was 0·08%. The variances found were smaller than those of the thermometer used. The temperature control system is good, although the time to attain a stable temperature is long.

*Imprecision*

Table 6 summarizes the results of the within-run and between-run imprecision studies. The within-run imprecision is acceptable for all the analytes assayed. The between-run imprecision is acceptable for all the analytes assayed except iron.

*Table 8. Relative inaccuracy with human sera on (y) Hitachi 717 and (x) comparison instrument: (1) Ultrolab-Aurora, (2) RA 1000 (n = 100).*

| | Comparison instrument | Regression analysis ($y = bx + a$) | | | | |
|---|---|---|---|---|---|---|
| | | Range | $b$ | (95% C.L.) | $a$ | (95% C.L.) |
| Glucose (mmol $l^{-1}$) | 2 | 4·1–19·4 | 0·98 | (0·92, 1·00) | −0·08 | (−0·19, 0·23) |
| Creatinine (μmol $l^{-1}$) | 2 | 51–900 | 0·78 | (0·75, 0·81) | 4·42 | (1·92, 7·50) |
| Iron (μmol $l^{-1}$) | 1 | 0·8–42·2 | 1·14 | (1·04, 1·25) | 0·00 | (−1·56, 1·18) |
| AST (U $l^{-1}$) | 2 | 8–577 | 0·86 | (0·82, 0·90) | −1·78 | (−3·21, −0·75) |
| ALP (U $l^{-1}$) | 1 | 56–649 | 1·07 | (1·03, 1·12) | −0·11 | (−6·70, 5·81) |
| Total protein (g $l^{-1}$) | 2 | 49–88 | 1·00 | (1·00, 1·00) | 1·50 | (1·50, 1·50) |
| Calcium (mmol $l^{-1}$) | 2 | 1·5–2·7 | 1·00 | (0·70, 1·00) | −0·15 | (−0·15, 0·58) |

*Specimen-independent carry-over*

In the study of the reagent probe carry-over, possible contamination of total protein with iron was found. Possible washing unit-related contamination in the sequence from creatinine to total protein was also detected.

*Sample-related carry-over*

The results are shown in table 7. The mean of the $k$ values obtained is less than 2·0% for all the analytes assayed except iron [3].

*Method comparison with patients' specimens*

The results of the regression study for each of the analytes evaluated are shown in table 8. The extreme values obtained for the slopes were 0·78 and 1·14 and those for the intercepts were −1·78 and 4·42.

The results reflect good agreement with the comparison instruments except for creatinine; we found ($p < 0{\cdot}05$) some proportional and constant differences between the two analytical methods.

### References

1. European Committee for Clinical Laboratory Standards, *Guidelines for the Evaluation of Analysers in Clinical Chemistry*, ECCLS Document, Vol. 3, No. 2, June 1986.

2. Comisión de Instrumentación del Comité Científico de la Sociedad Española de Química Clínica, *Protocolo de Evaluación de Analizadores Automáticos: Evaluación de los Módulos Analíticos*, Doc. E, ver. 3; Bol. Inf. SEQC, 1986, No. 34, pp. 3–17.

3. Broughton, P., *Journal of Automatic Chemistry* **6** (1984), 94.

4. Passing, H. and Bablok, W., *Journal of Clinical Chemistry and Clinical Biochemistry* **21** (1983), 709.

5. Passing, H. and Bablok, W., *Journal of Clinical Chemistry and Clinical Biochemistry*, **22** (1984), 431.

